# Picture quiz

**Published:** 2024-10-02

**Authors:** Victor H Hu

**Affiliations:** 1 Assistant Clinical Professor: International Centre for Eye Health, London School of Hygiene & Tropical Medicine and Consultant Ophthalmologist: Mid Cheshire NHS Hospitals, UK.

**This quiz is based on a real patient. Read the information, then use your knowledge and clinical skills to answer the questions. We suggest you use a separate sheet of paper, then compare your answers with those provided further down.**

A 21-year old man presents with a history of a red, sore, itchy right eye. Clinical pictures were taken (Figure 1 a and b). He reports having had itchy eyes for the past 5 years and he frequently rubs his eyes. He also has eczema, including on the periorbital skin.

**Figure 1 a and b:**
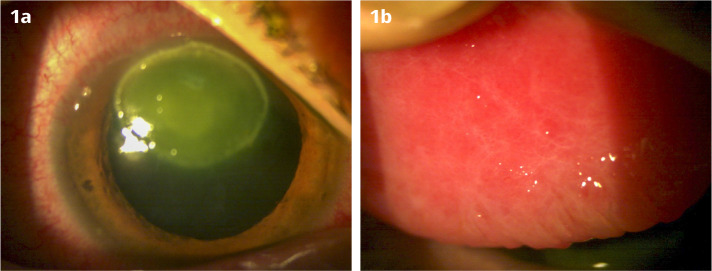
Initial presentation in a 21-year old man. UK

© VICTOR HU CC BY-NC-SA 4.0

**Question 1 What signs can you see on the images?**
**Question 2 What are your thoughts on what is going on with this patient?**
**Question 3 What is your management approach for this patient?**

## ANSWERS

**Figure 2:**
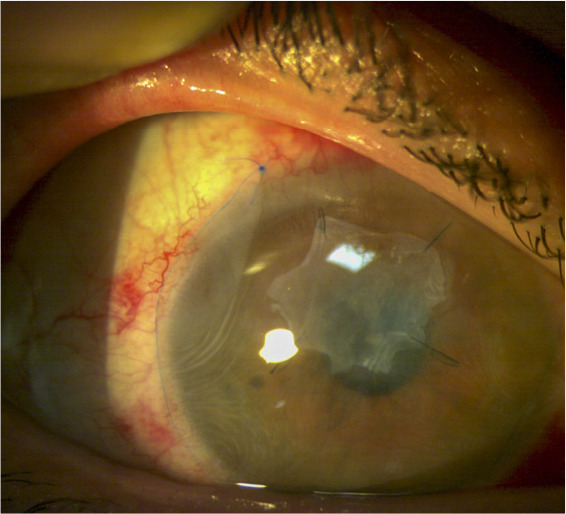
Amniotic membranes in place over the ulcer. UK

**1.** Conjunctival hyperaemia; a superior corneal oval-shaped epithelial defect with a white deposit at the base; marked tarsal conjunctival papillae with fine scarring.
**2.** This patient likely has severe allergic conjunctivitis. A more thorough history and examination are needed, but the presentation is consistent with atopic keratoconjunctivitis. This patient also has a shield ulcer on the cornea (something more commonly associated with vernal keratoconjunctivitis). An inflammatory plaque has formed at the base of the ulcer, formed of secreted proteins and mucin.
**3.** This patient needs urgent treatment for severe allergic conjunctivitis. A shield ulcer is a vision-threatening complication because corneal scarring, vascularisation, infection, or even perforation may develop.
  The eye shown in the image is likely to need many or all of the treatments listed below, and the other eye is likely to need some of them, depending on its clinical status. The patient should be monitored closely to assess his response to treatment, and treatment should be tapered gradually once there has been improvement. All drops should be preservative-free if possible, to avoid toxicity.
  - Conservative measures such as avoidance of triggering allergens; discussion about the importance of not rubbing the eyes, and use of cold compresses instead; lubricating eye drops; trial of oral antihistamine.
  - Antihistamine and mast cell stabiliser combination eye drops.
  - A course of steroid eye drops, initially very frequently and then tapering to minimise side-effects.
  - A topical calcineurin inhibitor, if available; either ciclosporin or tacrolimus.
  - Broad-spectrum antibiotic eye drops to prevent infection developing in the ulcer.
  - A supratarsal steroid injection. This can be considered immediately or after a few days, depending on the response to the other treatment.
  Shield ulcers often require surgical debridement, especially if there is a dense plaque at the base of the ulcer.^1^ This should be considered after 1–2 weeks if the above treatment is not successful. The procedure is done in theatre, with good anaesthesia, using a number 15 surgical blade or similar to debride the plaque down to clear cornea. If available, an excimer laser can be also used to perform a superficial keratectomy.^2^
  The addition of an amniotic membrane graft can help to reduce the inflammation and allow the cornea to re-epithelialise.^3^ Figure 2 shows the ulcer after the plaque was removed and with two layers of amniotic membrane in place: an inner layer over the area of shield ulcer and an outer one over the whole of the cornea and limbus.
  If available, amniotic membrane contact lenses can also be used as an alternative to sutured amniotic membrane. These are relatively quick to insert but provide only a single layer of amniotic membrane and tend to be expensive.

© VICTOR HU CC BY-NC-SA 4.0
